# Berberine Inhibits Human Hepatoma Cell Invasion without Cytotoxicity in Healthy Hepatocytes

**DOI:** 10.1371/journal.pone.0021416

**Published:** 2011-06-24

**Authors:** Bing Liu, Genshu Wang, Jie Yang, Xuediao Pan, Zhicheng Yang, Linquan Zang

**Affiliations:** 1 Department of Pharmacology, School of Pharmacy, Guangdong Pharmaceutical University, Guangzhou, People's Republic of China; 2 Department of Liver Surgery, the Third Affiliated Hospital, Sun Yat-Sen University, Guangzhou, People's Republic of China; 3 Department of Pharmacology, School of Pharmacy, Guangxi Medical University, Nanning, People's Republic of China; University of Barcelona, Spain

## Abstract

Conventional chemotherapy fails to cure metastatic hepatoma mainly due to its high hepatotoxicity. Many plant-derived agents have been accepted to effectively inhibit hepatoma cell invasion. However, the investigation that whether effectual plant-derived agents against invasive hepatoma cells exert unexpected cytotoxicity in healthy hepatocytes has been ignored. This study demonstrated that berberine exhibited significant cytotoxicity in HepG2 cells mainly through upregulation of reactive oxygen species (ROS) production but was ineffective in normal Chang liver cells. Berberine exerted anti-invasive effect on HepG2 cells through suppression of matrix metalloproteinase-9 (MMP-9) expression. Moreover, berberine could significantly inhibit the activity of PI3K-AKT and ERK pathways. Combination treatment of ERK pathway inhibitor PD98059 or AKT pathway inhibitor LY294002 and berberine could result in a synergistic reduction on MMP-9 expression along with an inhibition of cell invasion. Enhancement of ROS production by berberine had no influence on its suppressive effects on the activity of PI3K-AKT and ERK pathways, as well as MMP-9 expression and HepG2 cell invasion. In conclusion, our results suggest that berberine may be a potential alternative against invasive hepatoma cells through PI3K-AKT and ERK pathways-dependent downregulation of MMP-9 expression. This study also provides a previously neglected insight into the investigation of plant-derived agents-based therapy against tumor invasion with the consideration of damage to healthy cells.

## Introduction

Hepatoma is one of the most frequent and death-leading visceral neoplasms worldwide [1]. Most hepatoma patients have underlying hepatic dysfunction, which complicates safe administration of systemic therapy and conduction of trials of new agents [2].

Invasive phenotypes are fundamental components of malignant hepatoma, and thus become critical targets of anti-cancer agents development [3]. Most advanced hepatoma cells, which become progressively dedifferentiated, have high proliferative activity and progress to give rise to invasion and metastasis [4]. The most widely used agent against invasive hepatoma cells is doxorubicin, either as a single agent or in combination with other chemotherapeutics like cisplatin. However, this conventional chemotherapy has shown only a minimal survival advantage, as the hepatotoxicity of doxorubicin, which may undoubtedly aggratate hepatic dysfunction, remains a severe concern [5].

Many plant-derived agents with few adverse effects have been accepted as potential alternatives to the therapy for invasive hepatoma [6], [7], [8], [9]. However, most of these agents have failed to exert antiproliferative/cytotoxic effects on hepatoma cells within their suited anti-invasive doses [8], [9]. More importantly, to date, the investigation that whether effectual plant-derived agents against invasive hepatoma cells have unexpected cytotoxicity in healthy hepatocytes has been neglected.

Berberine, a clinically important natural isoquinoline alkaloid derived from Berberis species, has been reported to exhibit multiple pharmacological activities including anti-cancer effect [10]. A recent report indicated that berberine could induce hepatoma cell apoptosis through a mitochondria/caspases pathway while elicit no cytotoxic effects in healthy hepatocytes [11]. Yet the exact mechanism underlying the different effects of berberine on highly proliferative hepatoma cells and normal hepatocytes has not been fully elucidated. Specifically, berberine has gradually entered the limelight for its potentially therapeutic effect against invasion and metastasis of various lines of cancers such as glioma, lung cancer and nasopharyngeal carcinoma [12], [13], [14]. Very recently, berberine was firstly reported to inhibit melanoma cell migration, an essential step in invasion, by inhibition of COX-2, PGE_2_ and PGE2 receptors [15]. Nevertheless, no information about the action of berberine on invasive hepatoma cells has been addressed.

In this study, we explored the effects of berberine on malignant invasive phenotypes of HepG2 cells (a highly invasive human hepatoma cell line [16]). Our results demonstrate the critical component of ROS production in berberine-induced inconsistent cytotoxic effects on HepG2 cells and normal Chang liver cells. Specifically, berberine, without any cytotoxic effect on normal hepatocytes, inhibits HepG2 cell invasion through suppression of MMP-9 expression by concomitant inactivation of the PI3K-AKT and ERK pathways. The cytotoxic effect and the anti-invasive effect of berberine on HepG2 cells seem to be independently exerted.

## Materials and Methods

### Materials

Berberine was from Sigma (St. Louis, MO); PD98059 (MEK inhibitor) and LY294002 (PI3K inhibitor) were obtained from Merk. Cell culture reagents and DCFH-DA were obtained from Invitrogen. ERK 1/2 and AKT, the total and phosphorylated protein antibodies, MMP-9 antibody and horseradish peroxidase (HRP)-labeled anti-rabbit secondary antibody were purchased from Cell Signaling Technology (Boston, MA). All other reagents were from Sigma (St. Louis, MO) unless stated otherwise.

### Cell Lines and Cell Culture

The HepG2 cell line was originally obtained from the American Tissue Culture Collection (ATCC, USA). Cells were cultured at 37°C and 5% CO_2_ in Dulbecco's modified Eagle's medium (DMEM) supplemented with 10% fetal bovine serum (Gibco), 2 mM Glutamine, 1% non essential amino acids (NEAA) and 1% antibiotics (100 U/mL of penicillin and 100 µg/mL of streptomycin).

### MTT assay

Cell viability was determined by the MTT quantitative colorimetric assay, as previously reported [17]. 5×10^4^ cells in 100 µL of serum-free DMEM were seeded in 96-well and incubated with berberine at various concentration (0–80 µM) for 24 hours. Thereafter the medium was aspirated, and the cells were fixed with 0.2 mL of 10% cold TCA/per well at 4°C for 30 min, washed with deionized water, dried at room temperature overnight and incubated with MTT (0.5 mg/mL) for 4 hours. The viable cell number was directly proportional to the production of formazan solubilized with isopropanol, which could be measured spectrophotometrically at 563 nm.

### Assay for reactive oxygen species (ROS)

The intracellular generation of ROS was measured using DCFH-DA. The cell-permeable non-fluorescent dye penetrates into the cells and is hydrolyzed to DCFH by the cellular esterases. The probe (DCFH) is rapidly oxidized by ROS to the highly fluorescent compound 2′,7′-dichlorofluorescein (DCF). Cells were seeded in 6-well plates at 2×10^5^ cells/well and treated with or without berberine followed by incubated with 5 µM of DCFH-DA at 37°C for 15 min. After the preincubation, the cells were then washed twice with PBS, trypsinized, and resuspended in phosphate-buffered saline (PBS). At least 20,000 cells were acquired for each sample. The mean fluorescence intensity at 530 nm was assayed using a flow cytometer (Beckman Coulter, CA).

### Cell invasion assay

The effect of berberine on the invasiveness of HepG2 cells was determined using modified Boyden chambers consisting of Transwell (Corning Costar Corp, Cambridge, MA) with 8 µm pore size polycarbonate membrane filters precoated with 50 µL of Matrigel (1.25 mg/mL). During MTT assay, equal HepG2 cells (5×10^4^ cells) of the second group suspended in the serum-free DMEM of 100 µL in the presence or absence of berberine were seeded onto the upper chamber of Matrigel-coated filter inserts. Serum-containing DMEM (500 µL) was added to the lower chamber. After 24-hour incubation, filter inserts were removed from the wells. The cells on the upper surface of the filter were wiped with a cotton swab. Filters were fixed 4% paraformaldehyde for 30 minutes and stained with 0.1% crystal violet for 30 minutes, and then the invaded cells were determined as eight high-power fields of cells were counted in each well under an inverted microscope at 200× magnification. Invasion was calculated as the relative invasive score of treated group (invaded cell number/total cell number assayed by MTT represented by OD_570_) divided by that of control.

### Western blotting

Western blotting protocols were as in previous studies [18]. Briefly, cell lysates were separated by SDS/PAGE in 10% Tris-glycine gels and transferred to a NC membrane. For Western blot analysis of ERK1/2, phospho-ERK1/2, AKT and phosphor-AKT, blots were probed with their specific antibodies (diluted with 5% BSA to 1: 1000). Nonphosphorylated total ERK1/2 and AKT bands were chosen as loading control for MAPKs activation. For Western blot analysis of MMP-9, blots were probed with MMP-9 specific antibody (diluted with 5% BSA to 1: 500). Membranes were probed with horseradish peroxidase (HRP)–labeled anti-rabbit secondary antibody (diluted with 5% BSA to 1: 1000; all antibodies from Cell Signaling). Antibody binding was detected by enhanced enhanced chemiluminescence detection kit (ECL) (UK Amersham International plc). All Western blot exposures were in the linear range of detection, and the intensities of the resulting bands were quantified by Quantity One software on a GS-800 densitometer (Bio-Rad).

### SiRNA transfection

ene silencing by RNA interference (siRNA) was used to down-regulate MMP-9 expression in HepG2 cells. The siRNA specific for human MMP-9 was synthesized (Qiagen, Valencia, CA) against the target sequences for MMP-9: 5′-AACATCACCTATTGGATCCAAACTAC-3′, nucleotides 377 to 403 [19]. Nonsilencing siRNA (5′-AATTCTCCGAACGTGTCACGT-3′, Qiagen) with no homology to mammalian genes was used as the negative control [20]. Transfection of 7×10^5^ HepG2 cells with 0.1 µM of siRNA was done in a 66-mm Petri dish using 15 µL Lipofectamine 2000 (Invitrogen) according to the manufacturer's instructions.

### Statistical analysis

Data were statistically analyzed using Unpaired Student's t test at a significance level P value of <0.05 and are presented as means±SD, using Sigma Plot software (Jandel Scientific).

## Results

### Cytotoxic effect of berberine on HepG2 cells but not healthy hepatocytes

**Figure 1:**
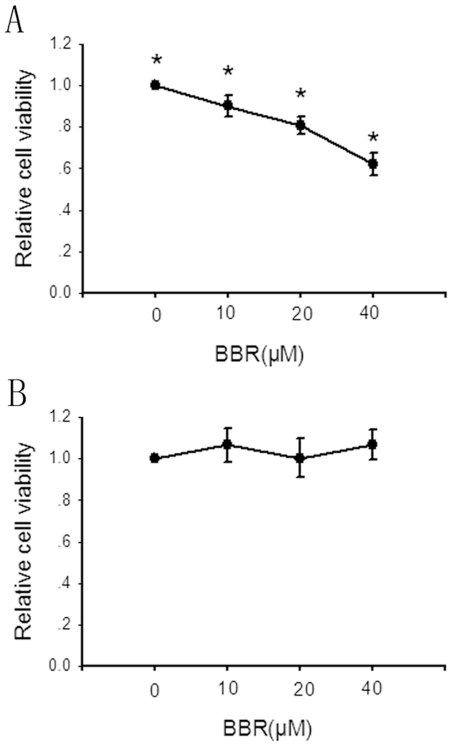
The effect of berberine (BBR) on the cell viability of HepG2 cells and Chang liver cells. HepG2 cells (A) or Chang liver cells (B) were treated with either 0.1% DMSO (as control) or berberine (10-40 µM) for 24 hours, and the proportion of surviving cells was measured by the MTT assay. Bars are mean±SD from six independent experiments. *Significantly different from control, P<0.05.

To determine the antiproliferative/cytotoxic effect of berberine on hepatoma cell line, in comparison with Chang liver cells, a non-tumor liver cell line, HepG2 cells (5×10^4^ cells) and Chang liver cells (5×10^4^ cells) were respectively suspended in 100 µL of DMEM and simultaneously seeded in 96-well plates. Then these cells were incubated in the absence or presence of increasing concentrations of berberine for 24 hours with the cytotoxicity of berberine measured by a standard MTT assay. As indicated in Figure 1A, the survival curve showed the dose-dependent cytotoxicity of berberine in HepG2 cells. After 24-hour of berberine (40 µM) treatment, cell viability was reduced by 40% approximately. In contrast, no marked antiproliferative/cytotoxic effects were seen in Chang liver cells under the exposure of same concentrations of berberine for 24 hours (Figure 1B).

### The dependence of berberine-induced cytotoxicity in HepG2 cells on upregulation of ROS production

**Figure 2:**
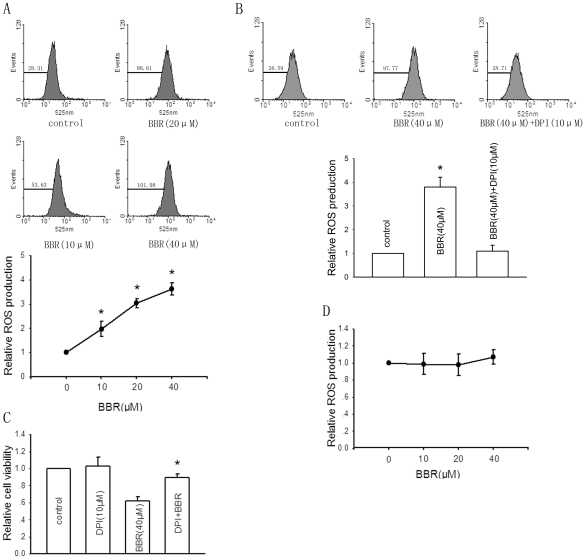
Berberine (BBR) induces cytotoxicity in HepG2 cells through upregulation of ROS production. A, Cells were treated with indicated concentrations of berberine for 24 hours. Intracellular ROS level was represented by the intensity of DCF fluorescence determined by flow cytometric analysis (left panel). The right graph represented the mean±SD of intensity of DCF fluorescence from five independent experiments. *Significantly different from DMSO control, P<0.05. B, The effect of pretreatment with 10 µM of DPI,a scavenger of ROS, on berberine-enhanced ROS production. Bars are mean±SD from four independent experiments. *Significantly different from DMSO control, P<0.05. C, The effect of pretreatment with 10 µM of DPI on berberine-induced cytotoxicity in HepG2 cells. Bars are mean±SD from five independent experiments. *Significantly different from only berberine (40 µM) treatment group, P<0.05. D, The effect of berberine of indicated concentrations on ROS production in Chang liver cells.

Excessive ROS production has been proposed as a vital role in induction of cell death by various agents [21]. To test the hypothesis that berberine-induced cytotoxicity in HepG2 cells is also initiated through upregulation of ROS level, we first used the DCFH-DA flow cytometry system to detect the effect of berberine on ROS production [22]. As shown in Figure 2A, treatment of HepG2 cells with berberine for 24 hours resulted in a dose-dependent increase in ROS generation compared with non-berberine-treated cells, which was demonstrated by the increase in intensity of DCF fluorescence. Pretreatment with DPI (10 µM), an inhibitor of NADPH oxidase [23], blocked the berberine-increased ROS production (Figure 2B). Next, we found that elimination of increased ROS by pretreatment with DPI (10 µM) reversed berberine-induced cytotoxic effect on HepG2 cells (Figure 2C). These results suggested that berberine exerted cytotoxic effect on HepG2 cells through enhancement of ROS production. On the contrary, berberine had no effect on ROS production in Chang liver cells (Figure 2D).

### Inhibitory effect of berberine on HepG2 cell invasion through suppressing MMP-9 expression

**Figure 3:**
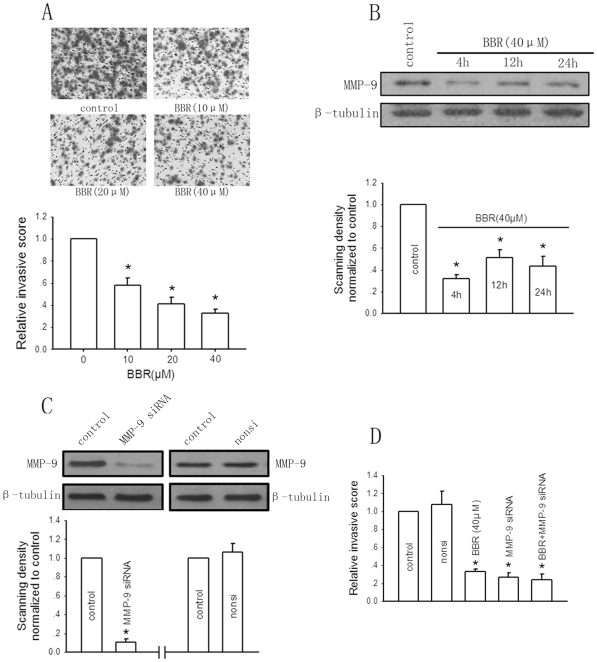
The MMP-9 expression-dependent anti-invasive effect of berberine (BBR) on HepG2 cells. A, The effect of berberine (10–40 µM) on HepG2 cell invasion was determined by invasion assay after 24-hour incubation. Bars are mean±SD from six independent experiments. *Significantly different from DMSO control, P<0.05. B, The effect of berberine at indicated times of exposure on MMP-9 expression. The protein level of MMP-9 was determined using the specific antibody and western blotting. Bar graphs are derived from densitometric scanning of the blots. Bars are mean±SD from four independent experiments. *Significantly different from DMSO control, P<0.05. C, Effects of MMP-9 siRNA and nonsilencing siRNA (nonsi) on MMP-9 expression determined by western blotting. Bar graphs are derived from densitometric scanning of the blots. Bars are mean±SD from three independent experiments. *Significantly different from DMSO control, P<0.05. D, The effect of MMP-9 siRNA and berberine on cell invasion. Bars are mean±SD from five-senven independent experiments. *Significantly different from DMSO control, P<0.05.

Berberine has been shown to exert inhibitory effect on invasion of multiple human cancer cells. To test whether berberine also has the same effect in hepatoma cells, HepG2 cell invasion with berberine treatment was analyzed by Matrigel invasion assay. Figure 3A showed that berberine administration led to concentration-dependent decrease in cell invasion after 24-hour incubation. Berberine (40 µM) diminished the invasive ability of HepG2 cells substantially up to 32.8% of the control.

To rule out the possibility that decreased cell invasion after berberine treatment might be caused by decreased total cell number, in fact, we plated cells at the same density and culture medium volume as in the MTT assay into transwell chambers for invasion assay (see the section‘Cell invasion assay’ of methods). At the same time, moreover, equal cells of berberine-treated and -untreated groups were plated to 96-well plates for total cell number assay (MTT) represented by OD_570_. The invasiveness of HepG2 cells was expressed by the invasive score (number of invaded cells/total cell number).

MMP-9 has been described to be closely participated in capsular infiltration in hepatoma cells [24]. The present study further investigated the effect of berberine on MMP-9 expression in HepG2 cells for determination of the mechanism for berberine-induced suppressive effect on cell invasion. Figure 3B showed that treatment of cells with berberine (40 µM) significantly suppressed MMP-9 expression and the decrease in MMP-9 level relative to that of non-berberine-treated group was approximately 40% after a 24-hour incubation period.

To confirm a causal link between berberine-mediated downregulation of MMP-9 expression and decreased invasion, the expression of MMP-9 was blocked by transfecting cells with MMP-9 siRNA. Figure 3C showed that siRNA to MMP-9 at the concentrations of 0.1 µM decreased MMP-9 expression by 89% as compared with control. The nonsilencing siRNA had no effect on MMP-9 expression. Both MMP-9 siRNA and the nonsilencing siRNA exerted no cytotoxic effect on HepG2 cells (data not shown). Knockdown of MMP-9 expression with MMP-9 siRNA resulted in a significant reduction of HepG2 cell invasion. Berberine significantly inhibited HepG2 cell invasion, but this effect was not seen when cells were pretreated with MMP-9 siRNA (Figure 3D). Altogether, these results suggest that berberine inhibits HepG2 cell invasion through suppression of MMP-9 expression.

### Involvement of the PI3K-AKT and ERK pathways in inhibitory effects of berberine on HepG2 cell invasion and MMP-9 expression

To investigate the mechanism for anti-invasive effect of berberine on HepG2 cells, we determined whether interfering with the PI3K-AKT and ERK pathways affected the inhibition of cell invasion and MMP-9 expression by berberine.

**Figure 4:**
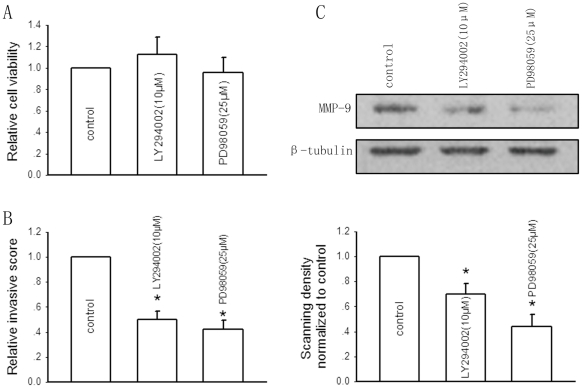
Effects of the PI3K-AKT and the ERK pathways on regulation of HepG2 cell invasion and MMP-9 expression. A, Effects of pretreatment of two specific inhibitors, the AKT inhibitor LY294002 (10 µM) and the ERK inhibitor PD98059 (25 µM) for 1 hour on the cell viability after 24-hour incubation assessed by MTT assay. The effects of pretreatment with LY294002 (10 µM) and PD98059 (25 µM) for 1 hour on HepG2 cell invasion (B) and MMP-9 expression (C) after 24-hour incubation. B, Bars are mean±SD from five independent experiments. *Significantly different from DMSO control, P<0.05. C, Bar graphs are derived from densitometric scanning of the blots. Bars are mean±SD from four independent experiments. *Significantly different from DMSO control, P<0.05.

We first determined the specific effect of the PI3K-AKT inhibitor LY294002 and ERK inhibitor PD98059. After 24-hour incubation, both LY294002 and PD98059 at the indicated concentrations had no impact on HepG2 cell growth as assessed by MTT assay (Figure 4A). Pretreatment with LY294002 (10 µM) and PD98059 (25 µM) for 1 hour significantly decreased the invasive ability of the cells, as the relative invasive score was reduced by near 50% and 58%, respectively (Figure 4B). Figure 4C showed that pretreatment with two inhibitors substantially downregulated MMP-9 expression by 31% and 56% respectively after 24-hour incubation, which suggested the critical contribution of PI3K-AKT and ERK pathways-dependent MMP-9 expression to the invasive phenotype of HepG2 cells.

To exclude the possibility that the PI3K-AKT and ERK pathways-dependent MMP-9 expression is restricted to hepatoma cells, experiments were also performed in normal Chang liver cells. Like HepG2 cells, PI3K-AKT and ERK pathways-dependent regulation of MMP-9 expression may also exist in normal Chang liver cells (See Text S1 and Figure S1, available online).

**Figure 5:**
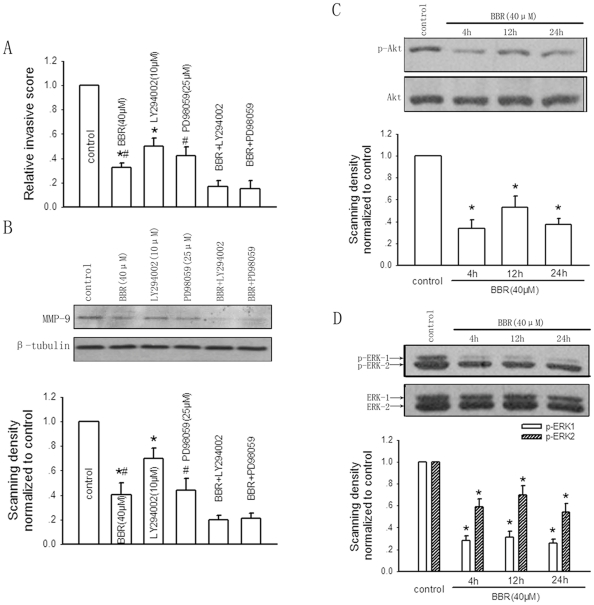
Involvement of the PI3K-AKT and ERK pathways in the inhibitory effect of berberine (BBR) on HepG2 cell invasion and MMP-9 expression. The effects of combination treatment of AKT inhibitor LY294002 (10 µM) or ERK inhibitor PD98059 (25 µM) with berberine on HepG2 cell invasion (A) and MMP-9 expression (B) after 24-hour incubation. Bar graphs are derived from densitometric scanning of the blots (B). Bars are mean±SD from four-seven independent experiments. *Significantly different from combination treatment of LY294002 (10 µM) and berberine (40 µM) group; #significantly different from combination treatment of PD98059 (25 µM) and berberine (40 µM) group, P<0.05. The effects of berberine (40 µM) on AKT (C) and ERK (D) activities represented as the levels of phosphorylated forms of AKT and ERK by western blotting at the indicated times of treatment. Bar graphs are derived from densitometric scanning of the blots. Bars are mean±SD from four independent experiments. *Significantly different from DMSO control, P<0.05.

Figure 5A illustrated that treatment of HepG2 cells with berberine (40 µM), LY294002 (10 µM) and PD98059 (25 µM) at the indicated time of incubation resulted in significant decrease in invasive ability of HepG2 cells, as the relative invasive score was reduced by approximately 67%, 50% and 42%, respectively. The combination treatment of LY294002 or PD98059 with berberine could even reduce the cell invasion by 83% or 85%, as compared with control. Furthermore, combination treatment of LY294002 or PD98059 with berberine could further downregulate the MMP-9 level compared with that of berberine treatment only (Figure 5B). Next, we determined the effect of berberine on AKT and ERK activities represented as the levels of phosphorylated forms of AKT and ERK by western blotting. Berberine (40 µM) significantly decreased the levels of p-AKT (Figure 5C) and p-ERK1/2 (Figure 5D) with no changes on total AKT and ERK levels under 4-, 12-, and 24-hour exposure, suggesting the suppressive effect of berberine on the PI3K-AKT and ERK pathways.

Thus, these results demonstrate that berberine inhibits the invasive ability of HepG2 cells through downregulation of MMP-9 expression by concomitant inactivation of the PI3K-AKT and ERK pathways.

### No involvement of upregulation of ROS production by berberine in its suppressive effects on the activity of PI3K-AKT and ERK pathways, as well as MMP-9 expression and HepG2 cell invasion

To explore whether the cytotoxic effect and the anti-invasive effect of berberine on HepG2 cells are independently exerted or have some crosslinks, we determined if enhancement of ROS production by berberine had some influence on its anti-invasive effect.

**Figure 6:**
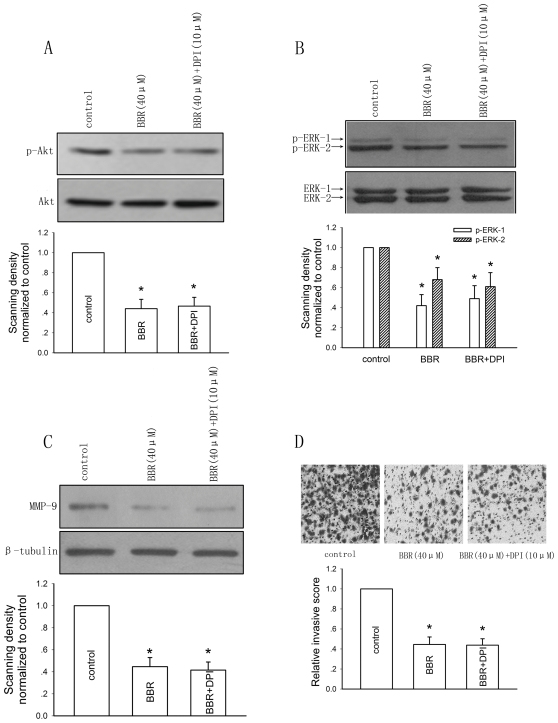
The effect of enhancement of ROS production by berberine (BBR) on its suppressive effects on the activity of PI3K-AKT and ERK pathways, as well as MMP-9 expression and HepG2 cell invasion. The effect of pretreatment with 10 µM of DPI on berberine-suppressed AKT (A) and ERK (B) activities represented as the levels of phosphorylated forms of AKT and ERK by western blotting after 24-hour incubation. Bar graphs are derived from densitometric scanning of the blots. Bars are mean±SD from three independent experiments. *Significantly different from DMSO control, P<0.05. C, The effect of pretreatment with 10 µM of DPI on berberine-suppressed MMP-9 expression after 24-hour incubation. Bar graphs are derived from densitometric scanning of the blots. Bars are mean±SD from three independent experiments. *Significantly different from DMSO control, P<0.05. D, The effect of pretreatment with 10 µM of DPI on berberine-suppressed HepG2 cell invasion after 24-hour incubation. Bars are mean±SD from five independent experiments. *Significantly different from DMSO control, P<0.05.

As shown in Figure 6, berberine administration significantly suppressed MMP-9 expression and invasion of HepG2 cells. Pretreatment with DPI had no influence on the suppressive effects of berberine on the activity of PI3K-AKT (Figure 6A) and ERK (Figure 6B) pathways, as well as MMP-9 expression (Figure 6C) and HepG2 cell invasion (Figure 6D). Thus, the cytotoxic effect and the anti-invasive effect of berberine on HepG2 cells seem to be independently exerted.

## Discussion

This study investigates the effect of berberine on viability and invasive capacity of HepG2 cells. Our results indicate that berberine selectively induces cell death in HepG2 cells while has no cytotoxicity in normal Chang liver cells. Specifically, we demonstrate that berberine, without cytotoxicity in normal hepatocytes, exerts suppressive effect on HepG2 cell invasion probably through concomitant inactivation of PI3K-AKT and ERK pathways, leading to downregulation of MMP-9 levels.

The present study demonstrates that upregulation of ROS production by berberine contributes to its induction of cell death in HepG2 cells. Furthermore, different effects of berberine on ROS production in HepG2 cells and healthy Chang liver cells can account for its inconsistent cytotoxic effects on the two types of cells. Recent data has demonstrated that cytotoxicity of berberine in HepG2 cells is derived from the induction of apoptosis probably through activation of caspase-3 and caspase-8 followed by a subsequent fall in the levels of BcL-XL, BcL-2 and Bid [11]. As caspase-3 and/or caspase-8-dependent pathway is shown to be required for commitment to ROS generation-triggered apoptosis by many agents [25], [26], we propose that ROS production by berberine may be the upstream event to procaspases activation and could be the critical initiator of HepG2 cell apoptotic death. In contrast, such induction of cell death by berberine is not present in normal hepatocytes mainly due to its no influence on ROS production in these cells. Moreover, the inconsistent cytotoxic effects of berberine were also reported to exist in prostate cancer cells and prostate epithelial cells [27]. Therefore, the resistance to berberine-induced oxidative stress of normal cells but not their corresponding neoplastic cells appears nonspecific to certain tissues.

Many important signaling pathways have been reported to be involved in berberine-induced suppressive effect on invasion of multiple tumor cells. ERK1/2 and p38 signaling pathways as well as FAK, IKK, NF-kappaB-mediated pathways have been shown to mediate the anti-invasive effect of berberine on MDA-MB-231 human breast cancer cells and human SCC-4 tongue squamous cancer cells, respectively [28], [29]. Besides, berberine has been also shown to exert inhibitory effect on tumor cell invasion through the reduction of Rho kinase-mediated ezrin phosphorylation at threonine 567 or inhibition of PKC-mediated signaling pathway [12], [14]. In the present study, our work demonstrates a previously unknown inhibitory effect on HepG2 cell invasion by berberine. Furthermore, we show that berberine inhibits HepG2 cell invasion through its suppressive effect on MMP-9 expression. Combination treatment of berberine with the AKT inhibitor LY294002 or the ERK inhibitor PD98059 results in a synergistic reduction of MMP-9 expression and invasive potential of HepG2 cells. These results indicate that concomitant inactivation of PI3K-AKT and ERK pathways-dependent decreased MMP-9 expression contributes to berberine-induced anti-invasive effect on HepG2 cells.

It is worth noting that mitochondria-dependent ROS generation has been confirmed to be associated with the expression of MMPs and tumor cell invasion [30]. However, although NADPH oxidase-dependent ROS generation is involved in cytoskeletal remodeling [31], extravasation and angiogenesis [32], there has been no direct evidence for its role in MMPs expression and invasion of tumor cells [33]. In our study, pretreatment with DPI blocked the berberine-increased ROS production (Fig. 2B), which suggests that enhanced ROS level by berberine is likely NADPH oxidase-dependent rather than mitochondria-dependent, as DPI is an inhibitor of NADPH oxidase [23]. DPI administration has no influence on the suppressive effects of berberine on the activity of PI3K-AKT and ERK pathways, as well as MMP-9 expression and HepG2 cell invasion (Fig. 6). Thus, it is suggested from our results that NADPH oxidase-dependent ROS generation may not exert any direct influence on MMP-9 expression and invasion of HepG2 cells. Moreover, the cytotoxic effect and the anti-invasive effect of berberine on HepG2 cells seem to be independently exerted.

The expression MMP-9 is regulated by the upstream promoter sequence, of which the activator protein-1 (AP-1) and nuclear factorκB (NF-κB) binding sites are centrally involved [34]. AP-1 and NF-κB are well accepted to be involved in many pathological processes including tumor cell migration and invasion [35]. Cheng et al. showed that NF-κB modulates the radiation-enhanced MMP-9 activity and cell invasion in HepG2 cells [36]. Chia-Jui Weng et al. reported that induction of MMP-9 expression by PMA and LAB treatment possibly through regulation of the AP-1 and NF-κB DNA-binding activities [16]. Thus, as AP-1 and NF-κB are the downstream targets of ERK and AKT pathways [16], [37], it is suggested from our results that berberine inhibits ERK and AKT pathways-dependent MMP-9 expression probably through resultant suppression of AP-1 and NF-κB activities.

MMP-9 and MMP-2 play critical roles in the degradation of type IV collagen, a major constituent of the basement membrane, and are closely related to the invasion and metastasis of various cancer cells [38], [39]. Specially for hepatoma cell invasion, MMP-9 has been shown to be more likely to participate in hepatoma cell invasion than MMP-2 for its destruction of tumor capsule [24]. This work showing that specific inhibition of MMP-9 expression by siRNA substantially suppresses the high invasive potential of HepG2 cells also suggests the decisive role of MMP-9 in basal hepatoma cell invasion.

Human hepatoma cells have been shown to exhibit high expression and enhanced activity of ERK and AKT [40], [41], [42]. Activation of the ERK and AKT pathways are considered to contribute to increased tumor aggressiveness, yet the exact mechanisms remain obscure [36]. In this study, our results showing the dependence of MMP-9 expression and invasive capacity of hepatoma cells on activity of AKT and ERK pathways can partly account for the high hepatoma cell aggressiveness. Besides, many studies have also linked the activity of PI3K-AKT and ERK pathways to MMP-9 expression in other tumor cell lines. O-charoenrat et al. showed that beta-cellulin induced MMP-9 production and invasion in head-and-neck squamous carcinoma cells through activation of EGFR, MAPK and AKT [43]. In ovarian cancer cells, Thant et al. suggested that both ERK and AKT were required for the fibronectin-dependent activation of MMP-9 secretion and the resultant cell invasiveness [44]. Thus, activation of AKT and ERK pathways-dependent MMP-9 expression may be widespread in tumor cells and contribute to tumor progression. Besides, the result that activation of ERK and PI3K-AKT pathways significantly increased the level of MMP-9 in Chang liver cells (See Figure S1B) suggests that AKT and ERK pathways-dependent MMP-9 expression may also exist in Chang liver cells and not be specific to tumor cells.

Currently berberine is administrated orally in clinical practice, for example, treatment of cardiac arrhythmia [45]. Research of the anti-tumor effect of berberine still remains preclinical. Numerous studies, including the present study, have shown that berberine inhibits cell growth and invasion of various tumor cell lines *in vitro* with a large concentrations ranging from 20 µM up to 300 µM [14], [29], [46]. However, pharmacokinetic studies in humans have shown that berberine is poorly absorbed, and difficult to maintain 40 µM plasma concentration after oral administration [45]. Alternatively, the preparation of the micro-emulsion formulation could be a potential approach to achieve a high *C*_max_ of berberine, as it can significantly increase the rate and extent of absorption [47]. Specifically, intravenous administration may be another potential alternative to achieve a high *C*_max_ in terms of anti-tumor effect [48] . Nevertheless, more works is definitely needed to translate the preclinical data into clinical practice and make our *in vitro* observation on the anti-tumor effect of berberine clinically relevant.

Our work has some limitations. It is not known to what extent the present principal finding can be generalized to cell types other than HepG2 cells and Chang liver cells examined in this study. Notwithstanding the limitation, our study does demonstrate the therapeutic potential of berberine against hepatoma invasion with the advantage of no unexpected cytotoxicity in healthy liver cells. Specifically, our study also provides a previously neglected insight into the investigation of plant-derived agents-based therapy against tumor invasion with the consideration of damage to healthy cells.

## Supporting Information

Figure S1 Effects of the PI3K-AKT and the ERK pathways on regulation of MMP-9 expression in normal Chang liver cells. A, The effects of pretreatment with LY294002 (10 µM) and PD98059 (25 µM) for 1 hour on MMP-9 expression of normal Chang liver cells after 24-hour incubation. B, The effects of pretreatment of Chang liver cells with 740 Y-P (20 µg/ml), a specific activator of PI3K, for 6 hours or enterostatin (100 nM), a nonspecific activator of ERK, for 1 hour on MMP-9 expression of normal Chang liver cells after 24-hour incubation. Bar graphs are derived from densitometric scanning of the blots. Bars are mean±SD from three-four independent experiments. *Significantly different from DMSO control, P<0.05.
(TIF)

Text S1 The PI3K-AKT and ERK pathways-dependent downregulation of MMP-9 expression exists in Chang liver cells.
(DOC)
