# Supplementary material for: Berberine Inhibits Human Hepatoma Cell Invasion without Cytotoxicity in Healthy Hepatocytes
Source: PLoS One. 2011 Jun 24;6(6):e21416. doi: 10.1371/journal.pone.0021416 (PMC3123339; doi:10.1371/journal.pone.0021416)
Supplement: Text S1 — The PI3K-AKT and ERK pathways-dependent downregulation of MMP-9 expression exists in Chang liver cells. (DOC) [file pone.0021416.s002.doc]

To explore whether the PI3K-AKT and ERK pathways-dependent downregulation of MMP-9 expression exists in normal Chang liver cells, we have performed additional experiments to detect MMP-9 expression by Western blotting under various conditions. Consistent with a previous study [1], we found that Chang liver cells showed an insignificant signal for MMP-9. Pretreatment of Chang liver cells with LY294002 (10 μM) or PD98059 (25 μM) for 1 hour did not change the MMP-9 level after 24-hour incubation (Figure S1A) cause activation of PI3K-AKT and ERK pathways is not obvious in normal Chang liver cells [2,3], the observation that inhibitors of these pathways exerted no effect on MMP-9 expression may not necessarily exclude the possible PI3K-AKT and ERK pathways-dependent downregulation of MMP-9 expression in Chang liver cells.

Therefore, we detected the MMP-9 level in Chang liver cells after activation of PI3K-AKT or ERK pathways with a specific activator of PI3K, a cell-permeable peptide 740 Y-P or a nonspecific activator of ERK, enterostatin. Pretreatment of Chang liver cells with 740 Y-P (20 μg/ml) for 6 hours [4] or enterostatin (100 nM) for 1 hour [5] significantly increased the levels of p-AKT or p-ERK (data not shown), as well as the level of MMP-9 after 24-hour incubation (Figure S1B). Together with another study showing that the X protein (HBx) of hepatitis B virus (HBV) induced MMP-9 expression through activation of ERK and PI3K-AKT pathways in Chang liver cells [6], it is suggested that PI3K-AKT and ERK pathways-dependent regulation of MMP-9 expression may also exist in normal Chang liver cells.

**References**

1. Kim JR, Kim CH (2004) Association of a high activity of matrix metalloproteinase-9 to low levels of tissue inhibitors of metalloproteinase-1 and -3 in human hepatitis B-viral hepatoma cells. Int J Biochem Cell Biol 36: 2293-2306.

2. Lin SB, Li CH, Lee SS, Kan LS (2003) Triterpene-enriched extracts from Ganoderma lucidum inhibit growth of hepatoma cells via suppressing protein kinase C, activating mitogen-activated protein kinases and G2-phase cell cycle arrest. Life Sci 72: 2381-2390.

3. Wu T, Leng J, Han C, Demetris AJ (2004) The cyclooxygenase-2 inhibitor celecoxib blocks phosphorylation of Akt and induces apoptosis in human cholangiocarcinoma cells. Mol Cancer Ther 3: 299-307.

4. Purdie KJ, Whitley GS, Johnstone AP, Cartwright JE (2002) Hepatocyte growth factor-induced endothelial cell motility is mediated by the upregulation of inducible nitric oxide synthase expression. Cardiovasc Res 54: 659-668.

5. Park M, Oh H, York DA (2009) Enterostatin affects cyclic AMP and ERK signaling pathways to regulate Agouti-related protein (AgRP) expression. Peptides 30: 181-190.

6. Chung TW, Lee YC, Kim CH (2004) Hepatitis B viral HBx induces matrix metalloproteinase-9 gene expression through activation of ERK and PI-3K/AKT pathways: involvement of invasive potential. FASEB J 18: 1123-1125.
